# Supplementary material for: Quercetin Induces Anticancer Activity by Upregulating Pro-NAG-1/GDF15 in Differentiated Thyroid Cancer Cells
Source: Cancers (Basel). 2021 Jun 16;13(12):3022. doi: 10.3390/cancers13123022 (PMC8233818; doi:10.3390/cancers13123022)
Supplement: Supplementary file 1 [file cancers-13-03022-s001.zip › cancers-1208166-supplementary.pdf]

cancers

MDPI

Supplementary Materials

Quercetin Induces Anticancer Activity by Upregulating Pro-NAG-1/GDF15 in Differentiated Thyroid Cancer Cells

Yukyung Hong, Jaehak Lee, Hyunjin Moon, Chang H. Ryu, Jungirl Seok, Yuh-Seog Jung, Junsun Ryu* and Seung J. Baek*

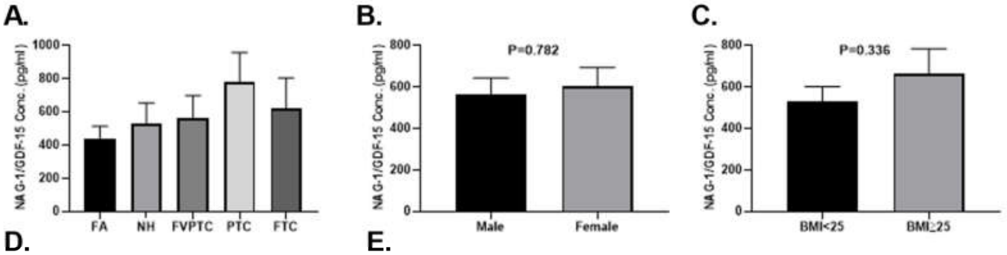

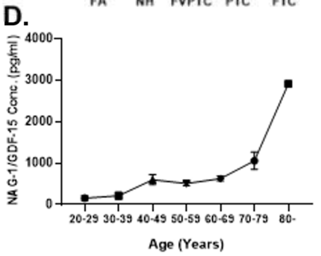

E.

	FA		NH		FVPTC		PTC		FTC	
**Female**	**mean(SD)**	**Range**	**mean(SD)**	**Range**	**mean(SD)**	**Range**	**mean(SD)**	**Range**	**mean(SD)**	**Range**
n	10		6		4		11		3	
age	48.1(10.24)	25-62	51.8(12.2)	31-60	49.3(8.7)	37-56	56.6(14.7)	26-82	46.7(24.99)	25-74
kg	63.5(11.07)	46.5-85	64.1(13)	49.8-83	59.6(9.7)	51.4-73.5	65.6(9.6)	52.2-81.2	60.5(85)	53.4-69.9
cm	162.8(6.59)	153.8-172.5	156.7(5.9)	146.4-164.2	156(4.4)	150.2-160.8	158.8(8)	147.4-175	157.9(8.71)	149.5-166.9
**Male**	**mean(SD)**	**Range**	**mean(SD)**	**Range**	**mean(SD)**	**Range**	**mean(SD)**	**Range**	**mean(SD)**	**Range**
n	5				3		3		4	
age	15.6	21-64			52.7(8.5)	44-61	67.7(5.7)	63-74	55.3(13.7)	38-72
kg	71.5(14.7)	59.7-95.6			70.1(3.1)	66.9-73	77.9(17.4)	67.6-98	79.1(8.3)	67.3-86.5
cm	170.8(9.6)	158.1-181			171.9(4.2)	167.7-176.1	170.9(1.4)	170-172.5	171.5(6.2)	162.9-177.4

Figure S1. NAG-1/GDF-15 concentration was increased by age. (A) ELISA assay was performed using plasma from thyroid cancer patients to measure concentration of NAG-1/GDF15. NAG-1/GDF15 level is not significantly different between thyroid cancer types. (B) There was no NAG-1/GDF-15 gender difference between female and male. (C) NAG-1/GDF15 concentrations did not differ between group with BMI above 25 and group with BMI below 25. (D) NAG-1/GDF15 level is increased with age as consistent with previous reports. (E) Patient information used in NAG-1 ELISA. FA, follicular adenoma; NH, nodular hyperplasia; FVPTC, follicular variant papillary thyroid carcinoma; PTC, papillary thyroid cancer; FTC, follicular thyroid cancer.

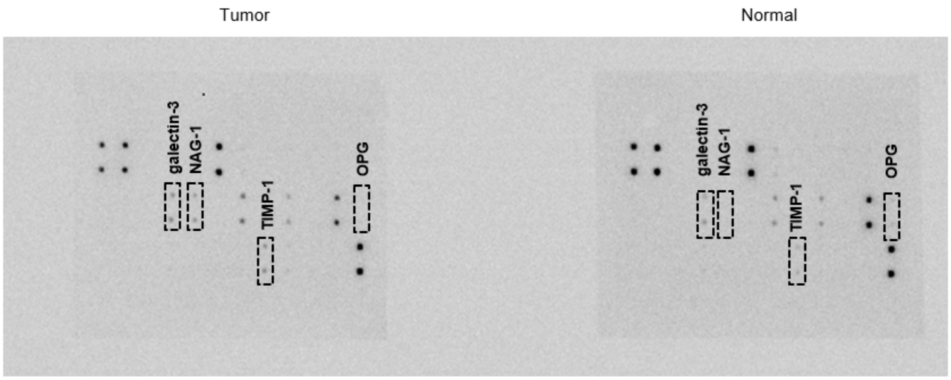

Figure S2. Uncropped Figure 1A.

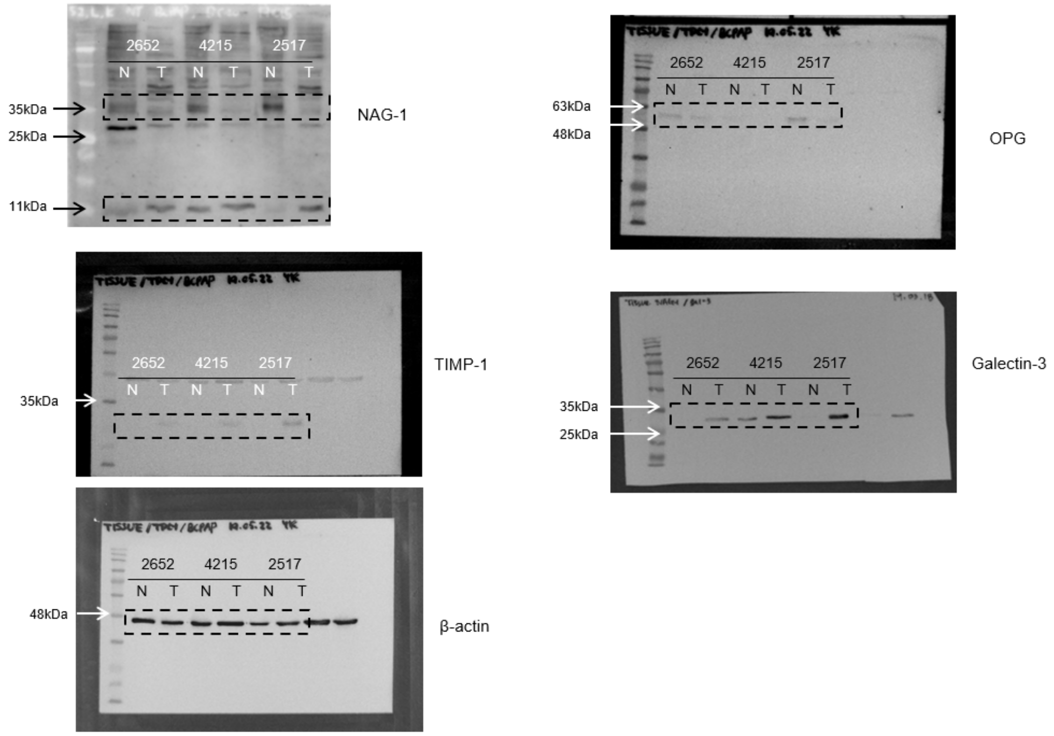

Figure S3. Uncropped Figure 1B.

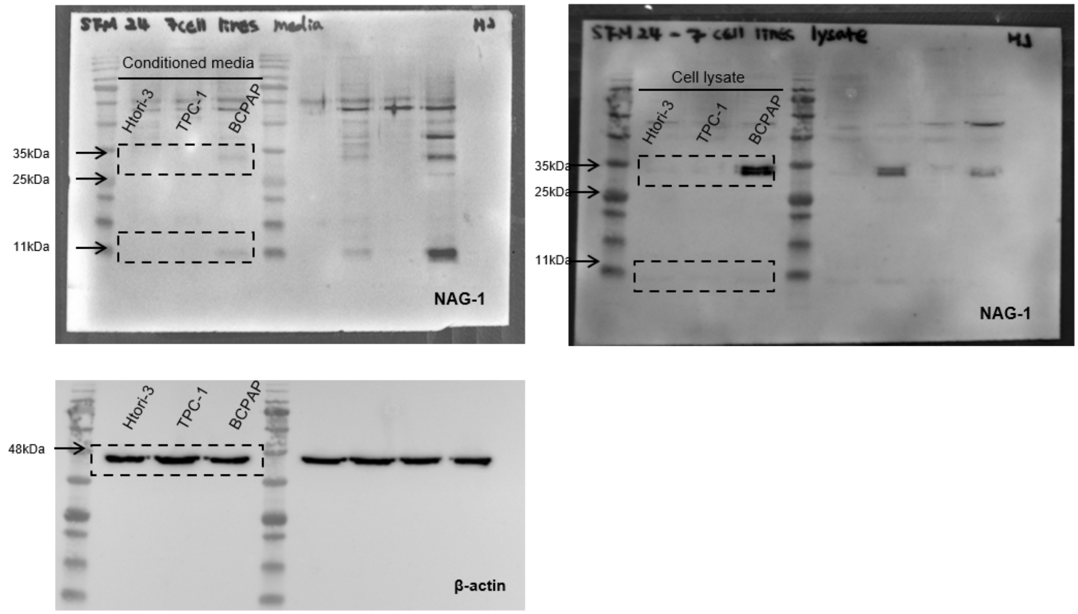

Figure S4. Uncropped Figure 2B.

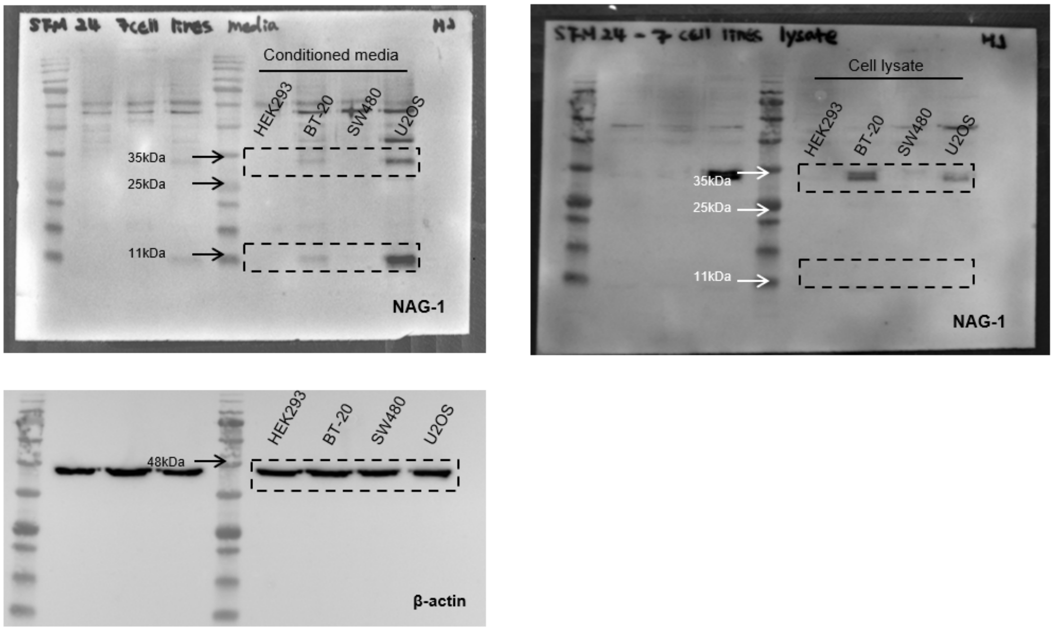

Figure S5. Uncropped Figure 2C.

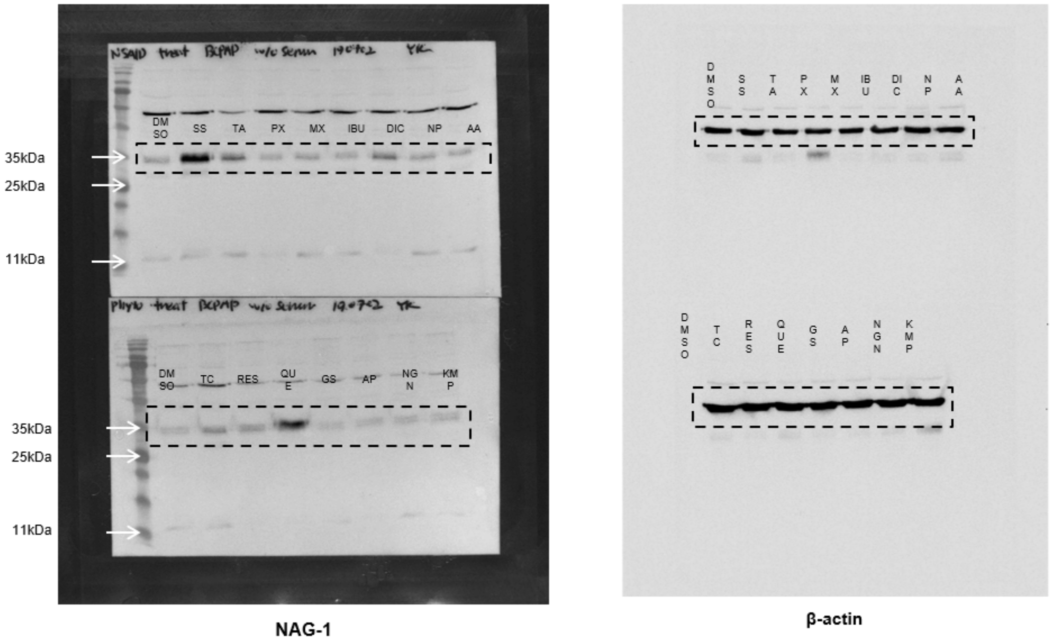

Figure S6. Uncropped Figure 3A, 3B.

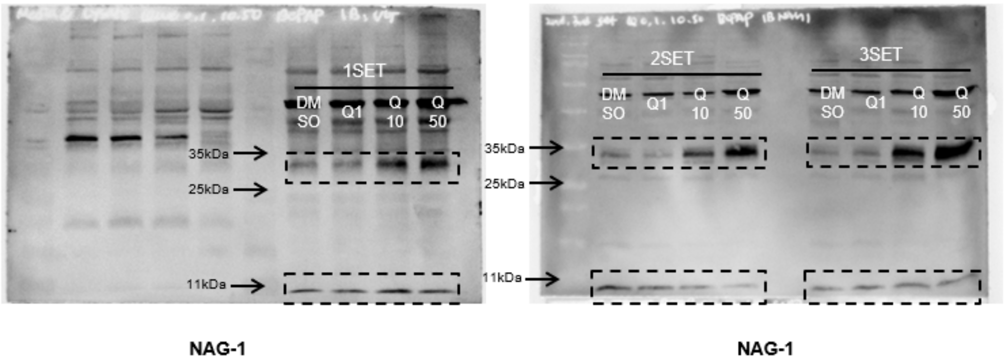

Figure S7. Uncropped Figure 3C.
